# Rethinking Pediatric Human-AI Interaction for Building Safer Digital Health Ecosystems

**DOI:** 10.7759/cureus.98369

**Published:** 2025-12-03

**Authors:** Hana Abbasian, Imeth Illamperuma

**Affiliations:** 1 Medical Education and Simulation, Center for Bioethics, Harvard Medical School, Boston, USA; 2 Medicine, McMaster University, Hamilton, CAN

**Keywords:** artificial intelligence (ai), digital health technologies, health policy and advocacy, machine learning tools, pediatric ethics

## Abstract

Artificial intelligence (AI) systems used in everyday digital spaces often rely on design assumptions shaped by adult patterns of reasoning, which creates specific interpretive gaps for younger users. This editorial examines how narrative-style outputs produced through epistemic automation can make probabilistic estimates appear more authoritative than intended for some adolescents. It also considers how technical opacity and model drift introduce shifts in system behavior that minors may misread as stable clinical logic, since there are few cues that distinguish computational changes from expert reasoning. When adolescents independently consult conversational agents or symptom-oriented tools, these interactions can influence clinical encounters without being systematically discussed. Therefore, this editorial outlines practical ways for clinicians to ask about AI-mediated information seeking and describes developmental design features, such as explicit uncertainty cues, layered explanations, and age-responsive prompting, that can reduce misinterpretation. Treating the pediatric digital ecosystem as a distinct design and regulatory setting allows for more precise alignment between algorithmic behavior, developmental cognition, and clinical practice.

## Editorial

The integration of artificial intelligence (AI) into pediatric life is accelerating in ways that exceed the conceptual tools clinicians use to evaluate risk. Children now routinely interface with algorithmic systems that function as informal health advisors [1]. These include generative conversational agents, predictive symptom models, adaptive learning platforms, and biomarker-inferring phone sensors [1,2]. Most of these systems were trained on datasets and design assumptions that reflect adult cognition [2]. This produces a structural mismatch between how the technology behaves and how a developing user interprets that behavior.

A central problem is the growing presence of epistemic automation, meaning the automation of judgment and explanation [3]. Many AI tools present probabilistic estimates as fluent narrative text. Emerging evidence shows heightened susceptibility to credibility inflation among younger users, where AI outputs are granted more authority than their underlying evidence justifies [2,4]. This is partly due to the emotional tone conveyed by the system, which young users may interpret as implicit social reassurance [4]. Pediatric care must articulate how these psychological mechanisms influence help-seeking behavior.

Technical opacity further complicates these challenges [3]. Many health-adjacent AI tools provide limited transparency around model updates and model drift, including how outputs change as the system retrains over time [2,3]. Minors are uniquely vulnerable to misinterpreting such outputs because they cannot distinguish between a system’s computational limits and a clinician’s reasoning process [4]. This interpretive gap widens as systems evolve without visible signals to users about when or why outputs have changed.

Current approaches and pediatric ethical frameworks rely heavily on parental mediation [5]. However, adolescents are autonomous digital actors [1,2], and they independently consult AI tools for questions related to sleep, nutrition, body changes, mood, and peer dynamics [1,4]. Without protective design patterns such as calibrated uncertainty displays, graded information depth, and age-responsive prompting, these interactions risk replacement of human guidance with algorithmic guidance without developmental alignment.

Clinicians may begin treating AI exposure as a routine part of pediatric assessment [4,5]. Asking adolescents about their use of symptom checkers, chatbots, wearable interpretations, and health apps can help contextualize presenting concerns. There is also a need for digital health literacy curricula that explicitly teach adolescents how to interrogate algorithmic claims. This includes understanding probabilistic reasoning, data provenance (where the training data came from), and the difference between correlation and clinical causation [3]. Establishing these developmental safeguards ensures that the digital ecosystem evolves in parallel with children’s cognitive capacities.

Finally, designers and regulators may consider the pediatric environment a distinct ecosystem that requires developmental protocols. This ecosystem must include algorithmic transparency summaries that are accessible to young readers, uniform labeling of AI-generated health content, and safety protocols that monitor for misinterpretation patterns over time.

If AI tools are becoming parallel health tools for young people, clinical practice must evolve to recognize and guide these interactions. The safety of pediatric digital health now depends on our ability to integrate technical architectures with developmental psychology and ethical governance, forming a coherent framework that respects the realities of how young users navigate digital health information. Progress toward this goal will depend on inclusive design standards, pediatric clinical practices that systematically account for AI exposure, and regulatory structures in alignment with children’s developmental needs.
